# Minimizing nocebo effects by conditioning with verbal suggestion: A randomized clinical trial in healthy humans

**DOI:** 10.1371/journal.pone.0182959

**Published:** 2017-09-14

**Authors:** Danielle J. P. Bartels, Antoinette I. M. van Laarhoven, Michiel Stroo, Kim Hijne, Kaya J. Peerdeman, A. Rogier T. Donders, Peter C. M. van de Kerkhof, Andrea W. M. Evers

**Affiliations:** 1 Unit Health, Medical and Neuropsychology, Institute of Psychology, Leiden University, Leiden, the Netherlands; 2 Leiden Institute for Brain and Cognition (LIBC), Leiden University, Leiden, the Netherlands; 3 Department for Health Evidence, Radboud University Medical Center, Nijmegen, the Netherlands; 4 Department of Dermatology, Radboud University Medical Center, Nijmegen, the Netherlands; 5 Department of Medical Psychology, Radboud University Medical Center, Nijmegen, the Netherlands; 6 Department of Psychiatry, Leiden University Medical Center, Leiden, the Netherlands; University of Auckland, NEW ZEALAND

## Abstract

Nocebo effects, i.e., adverse treatment effects which are induced by patients’ expectations, are known to contribute to the experience of physical symptoms such as pain and itch. A better understanding of how to minimize nocebo responses might eventually contribute to enhanced treatment effects. However, little is known about how to reduce nocebo effects. In the current randomized controlled study, we tested whether nocebo effects can be minimized by positive expectation induction with respect to electrical and histaminic itch stimuli. First, negative expectations about electrical itch stimuli were induced by verbal suggestion and conditioning (part 1: induction of nocebo effect). Second, participants were randomized to either the experimental group or one of the control groups (part 2: reversing nocebo effect). In the experimental group, positive expectations were induced by conditioning with verbal suggestion. In the control groups either the negative expectation induction was continued or an extinction procedure was applied. Afterwards, a histamine application test was conducted. Positive expectation induction resulted in a significantly smaller nocebo effect in comparison with both control groups. Mean change itch NRS scores showed that the nocebo effect was even reversed, indicating a placebo effect. Comparable effects were also found for histamine application. This study is the first to demonstrate that nocebo effects can be minimized and even reversed by conditioning with verbal suggestion. The results of the current study indicate that learning via counterconditioning and verbal suggestion represents a promising strategy for diminishing nocebo responses.

## Introduction

Nocebo effects, i.e., adverse treatment effects which are induced by patients’ expectations, play a central role in clinical practice [1, 2]. For example, patients receiving placebos in placebo-controlled clinical trials often report side effects similar to those experienced by patients receiving the active treatment [2]. These effects may be merely attributed to oral and written communication about potential adverse side effects in the informed consent procedure. Similarly, nocebo-induced side effects can also occur in response to an active treatment; this response can affect patients’ adherence and lead to withdrawal from necessary treatment [3]. Moreover, negative expectations regarding a certain treatment may reduce treatment effectiveness itself. For example, when patients are given an analgesic drug, positive expectations regarding its effects can double the effect, whereas negative expectations can completely abolish the analgesic effect [4]. A greater understanding of nocebo effects and how to diminish them is important for discovering ways of reducing their contribution to itch and other physical symptoms in clinical practice.

While most studies on nocebo effects derive from the field of pain, nocebo effects are also known to play a role in a range of other symptoms and conditions, such as gastrointestinal disorders, nausea, fatigue, allergic symptoms, and itch [5, 6][7]. Itch, like pain, is a common and severe symptom of several conditions and diseases, such as dermatological and systemic diseases, and can be a significant burden to patients, particularly when symptoms are chronic [8–11]. Chronic itch is associated with, for instance, lower quality of life, impairment of sleep, feelings of stigmatization, and depressive symptoms [9, 10]. Itch seems particularly susceptible to suggestion. This is demonstrated by the phenomenon of contagious itch–e.g., watching someone scratch himself can induce a sensation of itch in the perceiver [12, 13]–and by several recent studies demonstrating the role of nocebo effects on itch [14–17], by which nocebo effects might even be larger than in pain [14]. This makes itch a useful model to investigate the expectancy learning in nocebo effects.

With regard to expectancy learning in nocebo effects, the two expectation induction procedures that have been investigated most frequently are verbal suggestion and conditioning. Verbal suggestion consists of providing verbal or written information about clinical improvement or aggravation, such as potential side effects [18]. Conditioning, on the other hand, consists of repeatedly pairing a neutral stimulus (e.g., visual stimulus) with an active ingredient (e.g., increased pain stimulus), so that in time the neutral stimulus comes to elicit a similar response as the innate response (e.g., heightened experience of pain) [18]. Numerous studies have found evidence that verbal suggestion, conditioning and especially the combination of conditioning with verbal suggestion can induce nocebo effects on physical symptoms [15, 19–21]. As far as we know, only one study investigated whether nocebo-like effects can be reduced, using verbal suggestion procedures [22]. Reduction of nocebo effects, particularly induced via conditioning, has so far not been explored.

Changing of conditioned effects has been studied in fear and evaluative conditioning paradigms in particular [23–26]. Two main procedures that are used to change conditioned effects are extinction and counterconditioning. During extinction, a conditioned stimulus (CS) that was previously paired with e.g., a negative unconditioned stimulus (US) is now presented without the US. During counterconditioning, the CS-US pairing is still presented but the valence of the US is now opposite to the valence of the US with which it was previously paired (e.g., positive vs. negative) [23, 27–30]. Although extinction has been studied extensively, the results are mixed. Counterconditioning, on the other hand, has been investigated less frequently, but results show quite consistently that it can effectively change conditioned effects [25, 26, 31]. Counterconditioning has yet not been investigated with regard to nocebo (or placebo) effects, but could, in combination with verbal suggestion, prove an effective procedure to reduce nocebo effects.

In the present study we aimed to investigate whether conditioned nocebo responses to itch could be reduced by a positive expectation induction. Healthy participants were first exposed to a negative expectation induction (nocebo effect induction) by a procedure that combined conditioning and verbal suggestion regarding electrical itch stimuli. Then they were exposed to a positive expectation induction by a procedure that combined counterconditioning and verbal suggestion (placebo effect induction). In line with studies on counterconditioning e.g., [25, 32], control groups consisted of continued negative expectation inductions or an extinction procedure. It was hypothesized that the positive expectation induction would result in decreased itch in comparison with the two control groups. In addition, we exploratively tested the extent to which previously reduced nocebo effects would generalize to a different itch stimulus to assess external validity. Furthermore, it was explored whether psychological characteristics related to negative or positive outcome expectancies (e.g., worrying or optimism, respectively) were associated with (reversion of) nocebo responses [15, 33].

## Methods

### Ethics statement

The study was approved by the medical ethics committee of the Leiden University Medical Center in Leiden, the Netherlands (Commissie Medische Ethiek) and follows the rules stated in the Declaration of Helsinki. The study was registered retrospectively at the ISRCTN registry (registration code: ISRCTN 76895197), since this is a randomized experimental lab study in healthy individuals. The authors confirm that all ongoing and related trials for this intervention are registered. All participants gave written informed consent and were reimbursed for participation.

### Participants

In total 129 participants were included in this study. Exclusion criteria were severe physical morbidity (e.g., skin disease, diabetes mellitus, multiple sclerosis), psychiatric disorders (e.g., depression), chronic itch or pain complaints, diagnosis of histamine hypersensitivity, regular use of medication in the last 3 months, use of a pacemaker, and color blindness. All participants were of Dutch nationality, and were aged 18 years or older (mean age 20.25 ± 2.46 years; 78.7% were women.

### Design

**Fig 1:**
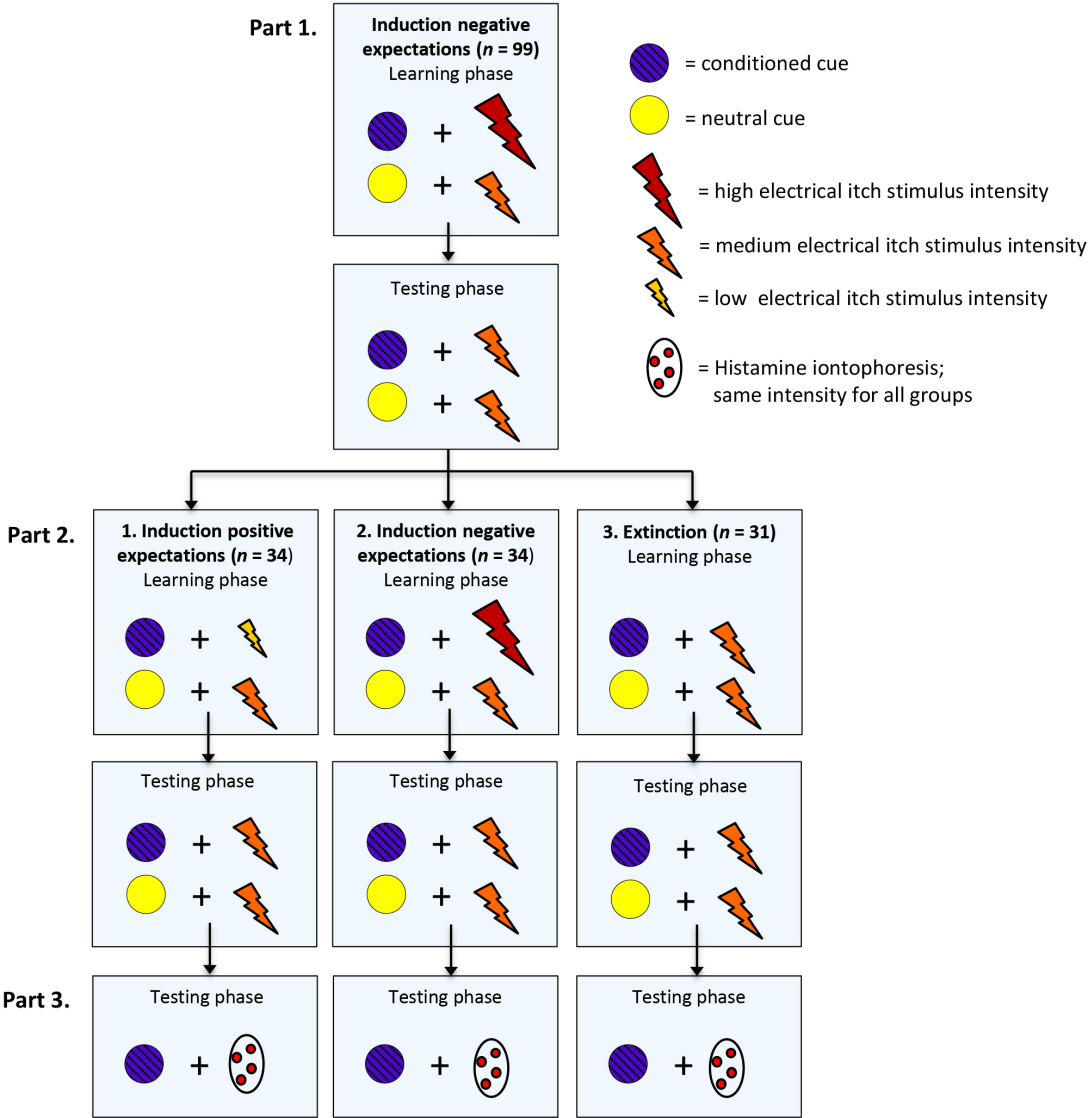
Experimental design. The study started with negative expectation induction: participants were told that the purple light (conditioned cue) indicated an increase in the itch stimulus, and that the yellow light (neutral cue) indicated no change in the itch stimulus. In accordance with the verbal suggestion, the purple and yellow lights were repeatedly paired with high and medium electrical itch stimulus intensities, respectively. Subsequently, participants were randomized over the three groups in which 1) positive expectations were induced; 2) continued negative expectations were induced; or 3) an extinction procedure was applied. In the learning phases verbal suggestion and conditioning procedures depended on the experimental group. In the testing phase the verbal suggestion corresponded to the verbal suggestion provided in the learning phase, while all participants received electrical itch stimuli of medium intensity. Next, generalization of reduced nocebo effects to histamine application was tested. The verbal suggestion corresponded to the verbal suggestion provided in part 2 and the purple light (conditioned cue) was displayed during the histamine application. The intensity of the histamine application was identical for all groups. Note that for half of the participants the conditioned cue was a purple light and the neutral cue a yellow light (like in this example); for the other half of the participants the conditioned cue was yellow and the neutral purple.

This study used a balanced (1:1:1) randomized, multi-arm parallel-group, single blind design. The study comprised three parts: in the first part, all participants received a negative expectation induction regarding electrical itch stimuli (induction nocebo effect; part 1); in the second part, participants were equally randomized over three experimental groups in which they either received a positive expectation induction (induction placebo effect; group 1), a continued negative expectation induction (induction nocebo effect; group 2), or an extinction procedure (extinction; group 3). In the third part, generalization of reduced nocebo effects to another itch stimulus, histamine iontophoresis, was tested. Fig 1. displays the experimental design.

### Materials

#### Itch induction

**Electrical stimulation.** Itch was induced by means of electrical stimulation using a constant current stimulator (Isolated Bipolar Constant Current Stimulator DS5, Digitimer, United Kingdom), and delivered to the inner side of the non-dominant wrist through two surface electrodes (for the detailed procedure see [15]). A third (sham) electrode was placed approximately 1 cm to the left from of the two active electrodes and attached to the back of the stimulator. Stimuli were applied at 50-Hz frequency with a pulse duration of 100 μs and at a continuously increasing current intensity (0.05 mA/s), up to a maximum current intensity of 5 mA. After each stimulus, participants verbally reported the level of itch, which they could express up to one decimal point, using a Numerical Rating Scale (NRS) ranging from 0.0 (no itch at all) to 10.0 (worst itch ever experienced). The NRS was attached below a computer screen in front of the participant.

**Histamine iontophoresis.** Histamine was applied by iontophoresis (Chattanooga Group, Hixson, TN, USA). A 0.3% diphosphate histamine solution was placed in an electrode (Chattanooga Ionto Ultra Electrode medium), which was placed on the dominant forearm (the forearm contralateral to the electrical itch stimulation), 2 cm distal to the lateral epicondyle of the humerus. The reference electrode was applied to the skin on the lateral side of the triceps brachial muscle. The histamine solution was delivered with a dose controller (Chattanooga ionto, Chattanooga Group, Hixson, TN, USA) for 2.5 minutes at a current level of 0.4 mA. The third (sham) electrode that was also applied during electrical stimulation, was placed approximately 1 cm to the left from of the two histamine electrodes and attached to the back of the electrical stimulator. Participants rated itch intensity on an NRS every 30 seconds during histamine application.

### Questionnaires

Screening questionnaires on demographic variables, diseases, and physical complaints were used to check participants for inclusion and exclusion criteria. In addition, several individual self-report questionnaires were used to assess the following psychological characteristics: *Optimism* (The Revised Life Orientation Test; [34]) total scale α = 0.66; *Hope* (The Dispositional Hope Scale; [35]) α = 0.81; *Worrying* (The Penn State Worry Questionnaire; [36]) α = 0.91; *Neuroticisms and Extraversion* (The Eysenck Personality Questionnaire; [37] neuroticism scale α = 0.79, α extraversion scale = 0.85; *Impulsivity* (The Baratt Impulsiveness Scale; [38] α = 0.84; *Self-efficacy* (The General Self-Efficacy scale; [39]) α = 0.82; *Negative affect* (The Hospital Anxiety and Depression Scale; [40]) total scale α = 0.79; *Future expectations* (The Future Expectations questionnaire; [41]) positive scale α = 0.86, negative scale α 0.76; *Positive and negative state affect* (Positive and Negative Affect Schedule; [42]) positive affect scale α = 0.73. The negative affect scale data were not analyzed due to strong floor effects (53% of participants reported minimum score); *State anxiety* (short version of the State-Trait Anxiety Inventory, State version; [43]) α = 0.69; *levels of itch*, *pain and fatigue* (Numerical rating scale (NRS); [44], participants reported the experienced intensity of the sensations on a NRS ranging from 0.0 (no itch/ pain/ fatigue at all) to 10.0 (worst itch /pain/ fatigue ever experienced). Furthermore, exit questions regarding the intensity of the stimuli and purpose of the study were used. All questionnaires were administered in Dutch. With the exception of the exit questionnaires, which were filled out on paper, all questionnaires were completed using Qualtrics (Qualtrics, Provo, Utah, United States).

### Procedure

The study was conducted at the Leiden University, Leiden, the Netherlands, from September 2014 to July 2015. Participants were recruited via an online recruitment system of Leiden University (Sona Systems, Tallinn, Estonia) and through flyers posted in the campus of the Leiden University, Leiden, the Netherlands. Participants were informed that the purpose of the study was to determine sensitivity to itch stimuli; the full purpose of the study was not revealed until after the experiment was finished. An online self-report questionnaire was used to screen participants for inclusion and exclusion criteria, and eligible participants were scheduled for an appointment. Next, participants filled out an additional online self-report questionnaires assessing personality traits.

All participants were asked to refrain from taking any medication, alcohol, and drugs for 24 hours before the test day, and from smoking cigarettes or drinking coffee, tea, cola, or energy drinks for two hours before testing. The experiment took place at a standard time (start at 11 am, duration ca. 5 hours and 30 minutes). First, all participants gave their written informed consent. Subsequently, baseline itch, pain, and fatigue were assessed using NRSs, and questionnaires on mood factors were administered. Before electrical stimulation, all participants held their hands in a warm water bath at about 32°C for 3 minutes in order to attain a comparable baseline wrist skin temperature among participants [15]. Next, the intensities of low, medium, and high stimuli were calibrated for each participant individually by gradually increasing the intensity of the electrical current with a ramping procedure (for the detailed procedure see [15]). The individually determined medium and high itch stimuli were used in part 1, and the low, medium and high itch stimuli were used in part 2 of this study.

In part 1, negative expectations regarding itch stimuli were induced in all participants by conditioning with verbal suggestion (part 1; induction of nocebo effect). Participants were told that they would receive a series of electrical itch stimuli with and without activation of a third electrode that influenced the itch intensity. This third electrode was actually never activated, since it was a sham electrode, serving as the ‘placebo’ in this experiment. Itch stimuli were accompanied by visual cues on a computer screen, i.e., purple and yellow colored circles. To control for effects of the colors, for half of the participants the conditioned cue was a purple circle and the neutral cue a yellow circle, and vice versa for the other half of the participants (an independent data manager generated an unpredictable random sequence via SPSS 23.0 for Windows; IBM SPSS Statistics, Chicago, Illinois, USA). Allocation to color was concealed by using sequentially numbered, opaque, envelopes that the experimenter opened just before the start of the learning phase in part 1 (1:1 allocation). The color was written on a folded sealed note. Participants were told: “A purple/ yellow light will signal the activation of the third electrode that induces an increase in the intensity of the itch stimulus, and the yellow/ purple light will indicate that the third electrode is turned off and will not change the intensity of the itch stimulus”. Conditioning was achieved by surreptitiously increasing the intensity of the itch stimuli on the conditioned trials relative to the neutral trials.

In part 2, a computer generated randomization list (generated by the independent data manager using SPSS 23.0 for Windows; stratified by sex; with a 1:1:1 allocation) was used to assign participants randomly to one of the three groups (which differed only in the verbal suggestion and conditioning procedure). Just before the learning phase of part 2 started, the experimenter opened a second sealed note in the envelope in which the experimental condition was revealed. Participants were unaware of randomization or differences between groups during the experiment.

In the positive expectation induction group (induction of placebo effect; group 1), expectations of low and medium levels of itch were now raised in the participants: “A purple/ yellow light will signal the activation of the third electrode, which will now induce a decrease in the intensity of the itch stimulus, and the yellow/ purple light will indicate that the third electrode is turned off and will not change the intensity of the itch stimulus”. In accordance with the verbal suggestion, conditioning was now achieved by surreptitiously decreasing the intensity of the itch stimuli on the conditioned trials relative to the neutral trials. In the negative expectation induction group (induction of nocebo effect; group 2), exactly the same procedure was applied as in the first part of the experiment. In the extinction group (extinction; group 3), no verbal suggestion was provided, i.e., participants were not given any information about the colored cues or stimulus intensity and all stimuli were applied at medium intensity.

In line with previous studies of conditioning in relation to nocebo and placebo effects on pain e.g.,[19, 45] and with a previous study of our own [15], the experimental session comprised two phases: a learning phase and a testing phase. The learning phase consisted of 16 trials in total: 10 conditioned trials with supposed activation of the third (sham) electrode, and 6 neutral trials without activation of the third electrode. These trials were presented in a quasi-random order for each participant i.e., there were no more than two conditioned trials in a row. The testing phase consisted of 8 trials in total: 4 conditioned trials and 4 neutral trials, again in quasi-random order, all followed by itch stimuli of medium intensity. Conditioning took place only in the learning phase, but verbal suggestions were also repeated in the testing phase; the suggestions were the same as in the learning phase.

Each trial consisted of a single itch stimulus, which was accompanied by a visual colored cue (purple or yellow) on a computer screen. To announce the start of a trial, every itch stimulus was preceded by a flashing colored cue of one second on the computer screen. Between each electrical itch stimulus applied in the learning and testing phases, there was a 2-minute interval, in which filler tasks (“find the differences” tasks, “word search puzzles”, and “Sudoku puzzles”) were given to diminish possible influence on subsequent stimuli of itch evoked by previously applied stimuli. The interval could be extended to a maximum of 4 minutes if the level of itch after 2 minutes was ≥2.0 on an NRS.

In part 3, histamine iontophoresis followed, using the same verbal suggestion as in part 2 and displaying the same conditioned cue on the computer screen during administration of the histamine itch stimulus using distinct electrodes. Participants were told that histamine would be applied to the skin through a light electrical current and that the skin could get red and thicker, similar to a mosquito bite. Before histamine was applied, participants indicated baseline levels of itch, pain, and fatigue. Then, participants were told: “During the itch stimulus, again a colored cue will be displayed at the computer screen. This will either be or a purple light, or a yellow light”. In the positive expectation induction group (induction of placebo effect; group 1), participants were told: “The purple/ yellow light will indicate a significant decrease in itch, and the yellow/ purple light will indicate that the itch remains unchanged”. In the negative expectation induction group (induction of nocebo effect; group 2), participants were told: “The purple/ yellow light will indicate a significant increase in itch, and the yellow/ purple light will indicate that the itch remains unchanged”. In the extinction group (extinction; group 3), no verbal suggestion was provided. In accordance with the electrical itch stimuli, the histamine itch stimulus was preceded by a flashing colored cue of one second on the computer screen. Even though participants were told that a purple or yellow light could be displayed, the color that was previously used for the conditioned trials (purple or yellow, depending on the randomization) was displayed. Moreover, the intensity of the histamine itch stimulus was, alike the testing phases in part 1 and 2 of the electrical stimulation equal for all groups.

Throughout the experiment participants were also videotaped in order to record scratching behavior and saliva was collected for DNA analyses (results will be reported elsewhere). The session was concluded with some questions regarding the perceived intensity of the itch stimuli and an open question to check whether participants were aware of the goal of the study.

During the test session, there were several standardized breaks. During the breaks, participants were provided a selected number of magazines to read, with neutral content (about nature and home decoration), and they were offered small snacks, Rooibos tea and water. Since participants sat down during the whole experiment, in the break after testing phase 1 participants took a short 2-minute walk within the research area of the university to stretch their legs, and used a home trainer in the lab at a slow pace for 5 minutes. There were no breaks between the learning and testing phases; the testing phases occurred immediately, without any signal.

### Statistical analyses

**Fig 2:**
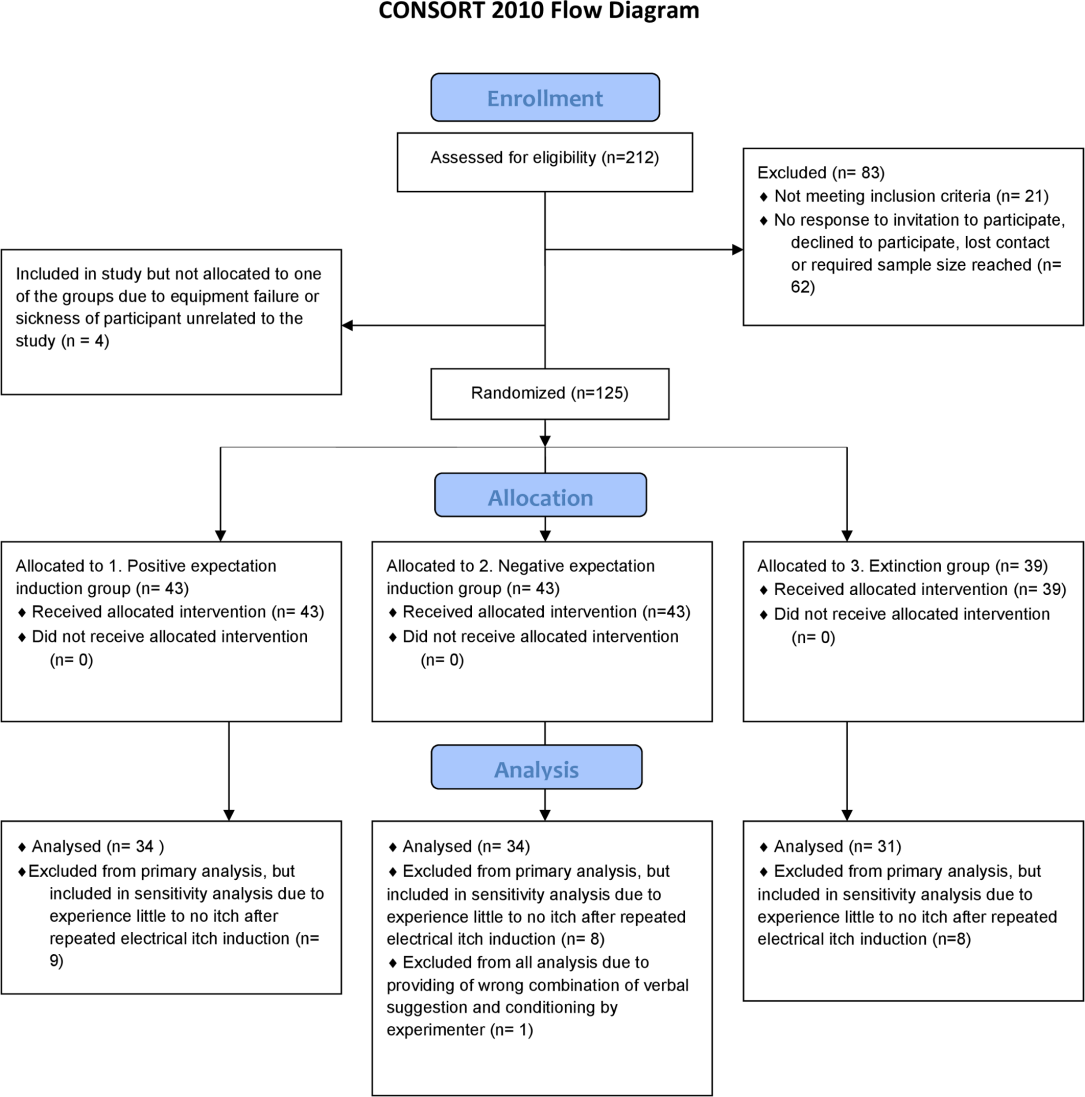
CONSORT flowchart.

All analyses were determined a priori in consultation with a statistician. The required sample size for the primary analysis was calculated with help from a statistician based on our previous study [15]. The analysis was approached in G*power 3.1 [46] as two two-tailed independent samples t-tests with Bonferroni correction. With an effect size *d* = 0.78, alpha *α* = 0.025 and a desired power of 0.80, this resulted in the largest required total sample size of 99 participants. In total 30 participants were excluded from data analysis on the basis of criteria determined in advance: for 1 participant, the experimenter provided a wrong combination of the conditioned cue with the verbal suggestion; 3 participants dropped out due to equipment failure; and 25 participants were excluded because they experienced little to no itch after repeated electrical itch induction (see also Fig 2. for more details on the number of participants at the different stages of the study). With the permission of the local ethical committee, it was decided to exclude all participants who rated the mean level of itch they experienced as < 1 itch on an NRS with regard to the itch stimuli associated with the neutral stimuli in the testing phase of part 2. These participants were replaced by randomly selected new participants. The statistical analyses were conducted over the participants who experienced ≥ 1 itch on an NRS (*n* = 99). However, sensitivity analyses were also carried out for all 124 participants who completed the study, of whom 25 had experienced < 1 itch on an NRS. All analyses were performed using SPSS 23.0 for Windows (IBM SPSS Statistics, Chicago, Illinois, USA). The nocebo effect was defined as the difference between the mean itch NRS scores associated with the four trials with the supposed activation of the third electrode (conditioned trials) and the four trials without the supposed activation of the third electrode (neutral trials) in the testing phase. A positive score indicated a nocebo effect.

Where the assumptions of the statistical tests (e.g., of normality) were violated, the data was transformed or non-parametric tests were used (if feasible). With regard to the nocebo effect in part 1 there was a problem with regard to normality of the data in the learning and testing phase. Although transformation did not result in normal distribution, parametric tests were reported, as non-parametric analyses obtained similar results.

Before conducting the main analysis, a paired samples t-test was performed to investigate whether there was a nocebo effect in the first part of the experiment by comparing the mean itch NRS score associated with the conditioned and neutral trials in the testing phase of part 1. As a manipulation check, the effectiveness of the negative expectation induction procedure during the learning phase was also assessed in an exploratory manner. To this end, a paired samples t-test was again performed as described above, exploring the difference in itch NRS scores between the conditioned and neutral trials in the learning phase of part 1.

Next, a univariate analysis of variance (ANOVA) was performed with *group* as between-subject factor in the second part of the experiment, in order to test the main hypothesis, i.e., that the *positive expectation induction group* would display a significantly smaller nocebo effect than the *control groups (negative expectation induction group* or *extinction group)*. Post hoc Dunnett tests were conducted to compare the *positive expectation induction group* with each of the *control groups*. As a manipulation check, the effectiveness of the expectation induction procedures during the learning phase was also assessed in an exploratory manner. Again, an ANOVA with post hoc Dunnett tests was performed, exploring the difference in change in itch NRS scores between the groups of part 2.

In order to exploratively assess generalization of reduced nocebo effects to histamine iontophoresis, first the mean itch NRS score was calculated for each participant (assessed at 0:30, 1:00, 1:30, 2:00, and 2:30 min). Next, an ANOVA was performed with *group* as between-subject factor and the mean itch NRS score during histamine application as dependent variable. Post hoc Dunnett tests were conducted to compare the *positive expectation induction group* with each of the *control groups*.

For exploratory purposes, Pearson correlation coefficients were calculated between the nocebo effects in the first as well as the second part (for each group separately) and questionnaire scores for psychological characteristics. For all analyses, the level of significance was set at *p*<0.05.

## Results

In part 1, negative expectations were induced in all participants (induction nocebo effect). In part 2 (reversing nocebo effect), randomization of the participants across the three groups resulted in a total of 34 participants in the *positive expectation induction group*, 34 participants in the *negative expectation induction group*, and 31 participants in the *extinction group*. The characteristics of age, gender, use of hormonal contraceptives, baseline levels of itch, pain and fatigue on the test day, and baseline levels of itch, pain and fatigue before histamine iontophoresis for the randomized participants were similar in the groups (see Table 1).

**Table 1:** Participant characteristics.

	1. Positive expectation induction	2. Negative expectation induction	3. Extinction
Age	20.4 ± 2.7	20.3 ± 2.7	19.9 ± 1.9
Male/female ratio %	26.5/73.5%	23.5/76.5%	19.4/80.6%
Hormonal contraceptives %	50.0%	50.0%	54.8%
Itch baseline test day NRS	0.6 ± 0.7	0.6 ± 0.7	0.6 ± 0.7
Pain baseline test day NRS	0.4 ± 0.9	0.4 ± 0.5	0.5 ± 0.7
Fatigue baseline test day NRS	2.4 ± 1.5	2.1 ± 1.5	2.1 ± 1.3
Itch baseline histamine NRS	1.0± 1.1	0.9 ± 1.1	0.6 ± 0.7
Pain baseline histamine NRS	0.7 ± 1.0	0.6 ± 0.6	0.6 ± 0.8
Fatigue baseline histamine NRS	3.6 ± 1.7	3.6 ± 1.3	3.8 ± 1.5

Characteristics of the participants in *the positive expectation induction group* (group 1; *n* = 34), *the negative expectation induction group* (group 2; *n* = 34), and *the extinction group* (group 3; *n* = 31).

### Induction of negative expectations (part 1)

#### Learning phase

As a manipulation check, the itch NRS scores evoked during the learning phase of part 1 were assessed. This involved the induction of negative expectations for all participants; both verbal suggestion and conditioning were applied. Table 2 displays the means (±*SD*) of the stimuli associated with the conditioned and neutral trials. A paired samples t-test revealed a significantly higher mean itch NRS score for the conditioned trials (*M* = 5.2, *SD* = 1.7) than for the neutral trials (*M* = 4.0, *SD* = 1.7) (*t*(98) = 12.55, *p*<0.001, *d* = 1.26). This result shows that the conditioning with verbal suggestion procedure was effective in inducing increased itch in the conditioned trials relative to the neutral trials.

**Table 2:** Means (±*SD)* for itch NRS scores in the learning and testing phase in part 1 (induction of negative expectations).

	Itch NRS scores (*M* ± *SD*)		
	Conditioned trials	Neutral trials	Change in itch score
*Learning phase*	5.2 ± 1.7	4.0 ± 1.7	1.2 ± 0.9
*Testing phase*	3.6 ± 1.9	3.2 ± 1.9	0.4 ± 0.8

Means (*M*) and standard deviations (*SD*) of the numerical rating scale (NRS) scores for itch and change itch NRS score (itch NRS score in conditioned trials minus neutral trials) in the learning phase and testing phase for the induction of negative expectations in part 1 (*n = 99*).

#### Testing phase

In the testing phase of part 1, all participants received the same stimuli, which were applied at medium intensity. Table 2 displays the means (±*SD*) of the itch NRS scores evoked by the stimuli associated with the conditioned and neutral trials. A paired samples t-test revealed a significantly higher mean itch NRS score for the conditioned trials (*M* = 3.6, *SD* = 1.9) than for the neutral trials (*M* = 3.2, *SD* = 1.8) (*t*(98) = 4.85, *p* < .001, *d* = 0.49), indicating a significant nocebo effect in part 1.

### Reversing nocebo effect (part 2)

#### Learning phase

As a manipulation check, the itch NRS scores evoked during the learning phase of part 2 were assessed. Depending on the group, positive or negative expectations were induced by conditioning with verbal suggestion or an extinction procedure was applied. Table 3 displays the mean (±*SD*) itch NRS scores evoked by the stimuli associated with the conditioned and neutral trials during the learning phases for each group. When we tested whether the mean change itch NRS score (conditioned trials minus neutral trials) would be smaller in the *positive expectation induction group* in than in the *control groups*, univariate analysis of variance (ANOVA) revealed a significant group effect (*F*(2,96) = 75.39, *p*<0.001, η_*p*_^*2*^ = 0.61). Post hoc Dunnett tests indicated a significantly larger itch NRS change score between the conditioned and neutral trials, for the *positive expectation induction group* (*M* = -1.5, *SD* = 1.0) as compared to the *negative expectation induction group* (*M* = 1.2, *SD* = 0.8) (*p* = < 0.001) as well as the *extinction group* (*M* = 0.7, *SD* = 1.0)(*p*<0.001). This result reveals that the positive conditioning procedure was effective in inducing decreased itch in the conditioned trials relative to the neutral trials, in comparison with the control groups.

**Table 3:** Means (*±SD)* for itch NRS scores in the learning phase for the different groups in part 2.

	Itch NRS scores (*M ± SD*)		
	Conditioned trials	Neutral trials	Change in itch score
***Group 1****—Positive expectation induction*	1.9 ± 1.5	3.3 ± 1.0	-1.5 ± 1.0
***Group 2*** *–Negative expectation induction*	4.2 ± 1.5	3.0 ± 1.5	1.2 ± 0.8
***Group 3*** *–Extinction*	3.8 ± 1.6	3.1 ± 1.6	0.7 ± 1.0

Means (*M*) and standard deviations (*SD*) of the numerical rating scale (NRS) scores for itch and for the change itch score (itch NRS score conditioned trials minus neutral trials) in the *positive expectation induction group* (group 1; *n* = 34), the *negative expectation induction group* (group 2; *n* = 34) and the *extinction group* (group 3; *n* = 31) in the learning phase of part 2.

#### Testing phase

**Fig 3:**
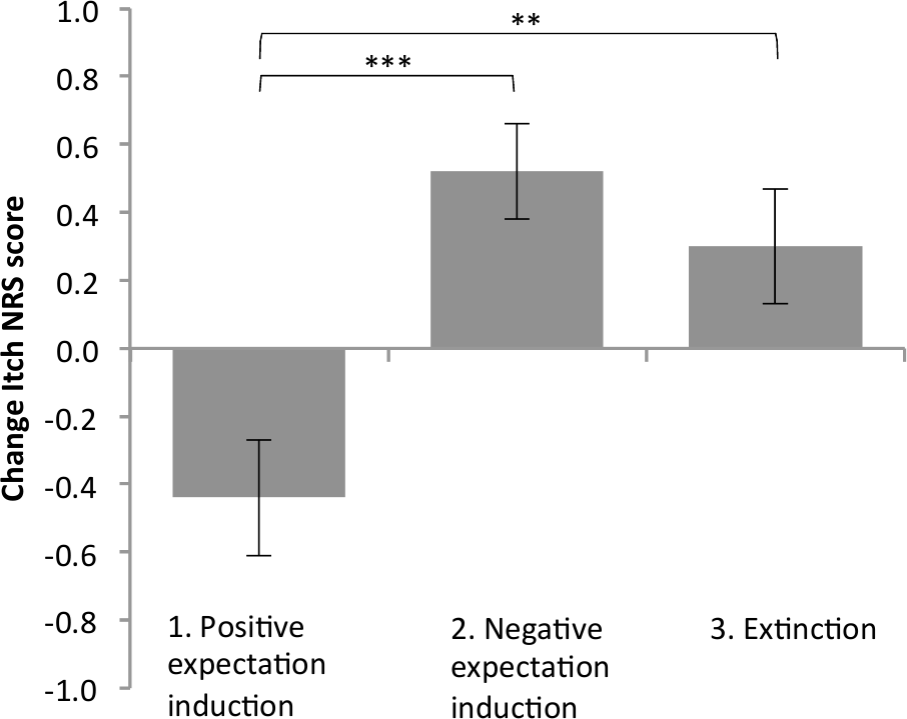
Nocebo effect. Means and standard error of the mean (error bars) of the numerical rating scale (NRS) itch scores for the nocebo effect (change in itch NRS score between the four conditioned and four neutral trials) of the different groups in the testing phase of part 2 (higher value indicates higher nocebo effect). The asterisks show the level of significance related to the post hoc Dunnett comparison (***p<0.001; **p<0.01).

In the testing phase of part 2, all participants received the same stimuli, which were applied at medium intensity. Table 4 displays the mean (±*SD*) itch NRS scores evoked by the stimuli associated with the conditioned and neutral trials during the testing phase for each group. The mean nocebo effect for each group is shown in Fig 3. When we tested the main hypothesis that the nocebo effect would be smaller in the *positive expectation induction group* than in the *control groups*, univariate ANOVA showed a significant difference in the magnitude of the nocebo effect in the various groups (*F*(2,96) = 9.93, *p*<0.001, *η*_*p*_^*2*^ = 0.17). Post hoc Dunnett tests comparing the experimental group with the control groups indicated a significantly smaller nocebo effect in *the positive expectation induction group* (*M* = -0.4, *SD* = 1.0) than in the *negative expectation induction group* (*M* = 0.5, *SD* = 0.8) (*p*<0.001) and the *extinction group* (*M* = 0.3, *SD* = 0.9) (*p* = 0.003) (See Fig 3.).

**Table 4:** Means (*±SD)* for itch NRS scores in the testing phase for the different groups in part 2.

	Itch NRS scores (*M* ± *SD*)		
	Conditioned trials	Neutral trials	Change in itch score
***Group 1****—Positive expectation induction*	2.4 ± 1.5	2.9 ± 1.5	-0.4 ± 1.0
***Group 2*** *–negative expectation induction*	3.4 ± 1.7	2.9 ± 1.9	0.5 ± 0.8
***Group 3*** *–Extinction*	2.9 ± 1.9	2.6 ± 1.9	0.3 ± 0.9

Means (*M*) and standard deviations (*SD*) of the numerical rating scale (NRS) scores for itch and for the change in itch score (itch NRS score conditioned trials minus neutral trials) in the *positive expectation induction group* (group 1; *n* = 34), the *negative expectation induction group* (group 2; *n* = 34) and the *extinction group* (group 3; *n* = 31) in the testing phase of part 2.

### Generalization of nocebo effects to histamine iontophoresis

When we explored whether the reduced nocebo effect generalized to the histamine stimulus, an ANOVA showed a significant main effect for the groups with regard to the mean itch NRS score during histamine application (*F*(2,96) = 5.293, *p*<0.01, η_*p*_^*2*^ = 0.10). Post hoc Dunnett tests comparing the experimental group with the control groups indicated significantly lower itch NRS scores in *the positive expectation induction group* (*M* = 5.7, *SD* = 0.3) than in the *negative expectation induction group* (*M* = 6.8, *SD* = 0.3) (*p*<0.01) and marginally significant itch NRS scores in the *positive expectation induction group* than in the *extinction group* (*M* = 6.5, *SD* = 0.3) (*p* = 0.058).

### Psychological characteristics

When we calculated correlation coefficients between the nocebo effect in part 1 (induction negative expectations) and psychological characteristics, no significant correlations were found. Similarly, when we calculated correlations coefficients between the psychological characteristics and the nocebo effect in part 2 for each group separately (reversing nocebo effect), no significant correlations were found.

### Sensitivity analyses

Sensitivity analyses were conducted regarding the main analysis to assess the influence of excluding the data of 25 participants who experienced little to no itch after repeated electrical itch induction (< 1 itch on an NRS, *see* methods, *statistical analyses*). When all 124 participants who completed the study were included, similar effects were found. More specifically, when we investigated whether there was a significant nocebo effect in the testing phase of part 1, a paired samples t-test revealed a significantly higher mean itch NRS score for the conditioned trials (*M* = 3.2, *SD* = 2.0) than for the neutral trials (*M* = 2.8, *SD* = 1.9) (*t*(123) = 5.45, *p* < .001, *d* = 0.96). This indicates that the nocebo effect in part 1 was significant. When we tested the main hypothesis that, if all 124 participants were included, the nocebo effect in part 2 would be smaller in *the positive expectation induction group* than in the control groups, univariate ANOVA showed a significant difference in the magnitude of the nocebo effect in the various groups (*F*(2, 121) = 12.23, *p* < 0.001, *η*_*p*_^*2*^ = 0.17). Post hoc Dunnett tests comparing the experimental group with the control groups indicated a significantly smaller nocebo effect in the *positive expectation induction group* (*n* = 43) (*M* = -0.4, *SD* = 0.9) than the *negative expectation induction group* (*n* = 42) (*M* = 0.5, *SD* = 0.8) (*p* < 0.001) and in the *extinction group* (*n* = 39) (*M* = 0.3, *SD* = 0.9) (*p* < 0.01).

## Discussion

The present study demonstrates, for the first time, that nocebo effects induced by conditioning with verbal suggestion can be minimized and even reversed by positive expectation induction by means of counterconditioning with verbal suggestion. Participants who received the positive expectation induction experienced significantly less itch than participants in the control groups, who received either continued negative expectation induction or an extinction procedure. Moreover, these results generalized to a second itch stimulus. These results demonstrate that a single session of counterconditioning with verbal suggestion is sufficient to reverse previously induced nocebo effects and elicit placebo effects.

In line with our previous studies on nocebo effects on itch [14, 15], exposing the participants to negative expectations (i.e., expectations for high levels of itch) regarding the conditioned trials, resulted in significantly higher levels of itch in response to the conditioned trials than to the neutral trials; this indicates that the nocebo induction was successful. This finding replicates results from a previous study [15], in which we demonstrated that nocebo effects on itch can be induced by the combination of conditioning and verbal suggestion. This is in accordance with studies on other physical symptoms like pain [19–21]. Furthermore, the result is consistent with previous studies that demonstrate that verbal suggestion alone can induce nocebo effects, or nocebo-like effects, on itch [14, 16, 47, 48]. The current study not only replicates the finding that nocebo effects on itch can be induced by conditioning and verbal suggestion [15], but also extends this by demonstrating that nocebo effects can be reversed. To the best of our knowledge, this is the first time that this has been investigated.

Positive expectation induction in the conditioned trials resulted in a significantly smaller nocebo effect than in the control groups, in which negative expectations were induced or an extinction procedure was applied. Moreover, the nocebo effect after positive expectation induction even demonstrated a significant placebo effect. Additional support for these findings was found in the learning phase, in which there was a similar pattern of changes in itch scores in response to conditioned and neutral trials. This finding extends results of a recent study on nocebo-like effects induced by verbal suggestions, which provided some initial indications that positively framed information regarding the health effects of wind turbine sound can dilute or even reverse the effects of negative expectations [22]. The successful reversal of the nocebo effect on itch by means of a counterconditioning procedure is consistent with a large body of research that shows that counterconditioning is an effective way of changing learned behavior in, for example, fear and evaluative conditioning paradigms [23–25, 32]. Furthermore, the finding that counterconditioning by inducing positive expectations was more effective than an extinction procedure in reversing the nocebo effect is also in accordance with conditioning studies that indicate that counterconditioning might be more effective than extinction in changing conditioned effects [23–25, 32].

In the current study, the extinction procedure did not significantly reduce nocebo effects. This is in line with previous studies on pain showing that nocebo effects eventually decrease but often do not fully eliminate the learned behavior, especially when a high number of conditioning trials is used [19, 45, 49]. Since in these studies and the current one the number of extinction trials was limited to a maximum of 10, it is currently unknown whether nocebo effects might be extinguished after more extinction trials or after several days. Similarly, evaluative conditioned effects seem less sensitive to extinction than conditioned fear responses, which often do become extinct after extinction trials [19, 45, 49]. In evaluative conditioning this is explained by the fact that the nonoccurrence of the US disconfirms the predictive value of the CS, but still evokes the representation of the US with the accompanying evaluation [31, 32]. More research is needed to establish whether similar processes could play a role in nocebo effects.

We found indications that the reduced nocebo effect generalized to a second, different itch stimulus i.e., histamine iontophoresis. The demonstration of possible generalization to other stimuli lends weight to the effectiveness of the counterconditioning with verbal suggestion procedure for the reduction of nocebo effects. However, future research should investigate whether this generalization is still effective without repeating verbal suggestions, as it was applied in our study before the histamine application. Moreover, this finding supports the external validity of the counterconditioning with verbal suggestion paradigm employed in this experiment. Therefore, also for other physical sensations like pain, it would be highly relevant to investigate the reversibility of nocebo effects, to get insight into expectancy learning in reversing nocebo effects across different sensations.

In the present study we did not find any significant correlations with the psychological characteristics examined. Previous studies regarding nocebo effects on itch have found indications for a role of psychological characteristics in relation to negative outcome expectancies, like worrying or negative affect, however research is extremely scarce [50]. Moreover, in a recent study by our research group, indications were found that one’s cognitive schemas regarding specificity and valence of memories and expectations regarding itch are related to placebo responding on itch, i.e., participants who were more specific in their memories regarding itch and who had less negative itch-related expectations for the future were more likely to be placebo itch responders [33]. Future research should further investigate the determinants of (reversing) nocebo responses, like individual differences in psychological characteristics in relation to negative outcome expectancies and cognitive schemas regarding memories and expectations.

Several implications for future research and clinical practice should be considered. First, the counterconditioning with verbal suggestion paradigm could possibly be applied to other experimental models of itch, like mechanical itch stimuli, to study different itch pathways that are relevant for different types of pruritus that can be seen in clinical practice [51, 52]. Second, it remains to be established whether these findings in healthy participants can be generalized to patients in a clinical setting. Two studies regarding contagious itch suggest that patients with chronic itch complaints might respond more strongly to visual or audiovisual itch cues than healthy controls [13, 53]. Additionally, several neuroimaging studies demonstrate differences in brain activation when itch is experimentally induced in patients versus in healthy controls [54, 55], emphasizing the need to study the placebo and nocebo effects on itch separately for healthy controls and patients. Furthermore, future research should investigate how experimental conditioning paradigms can be used in clinical practice. For example pharmacotherapeutic conditioning designs regarding itch medication, aimed at reducing the dose of medication could be examined. For example, a related format has been used in a study in patients with allergic rhinitis, in which an H1 receptor antagonist was conditioned with a novel-tasting drink, and in the testing phase replaced by a placebo with the drink. Patients reported less subjective symptoms and showed a reduced skin response to the skin prick test when administering the drink along with a placebo pill [6]. Future research could set up a similar design with reducing the dosage of itch medication to diminish possible side effects while the therapeutic benefits of the medication are preserved [56]. Minimizing possible nocebo effects could be an important ingredient of individually tailored care interventions for chronic somatic conditions [11, 57, 58]. This may be particularly important for patients with negative expectations regarding the given treatment, for example for patients with negative treatment experiences or certain personality characteristics related to negative treatment outcomes (e.g., worrying), or for patients who are excessively afraid of side effects [1, 50, 59, 60].

A possible limitation of this study is that reversal of the nocebo effect was tested in a single session. It would be highly relevant to test whether the reversed nocebo effect, i.e., placebo effect, remains on subsequent days, whether it extinguishes, or whether the nocebo effect recurs. Furthermore, we did not investigate the influence of the filler tasks provided between the electrical itch stimuli. Although we selected different tasks that were in general not too challenging and as neutral as possible, we cannot exclude a possible influence on for example mood, which can vary between individual participants. In addition, we did not assess participants’ expectations over the course of the study, so we cannot exclude the possibility that factors other than expectancy learning might be responsible for the effects found in this study. Moreover, assessing participants’ expectations in future studies would provide valuable data on how the induction of negative and subsequently of positive expectations affects patients’ expectations overall, and on the extent to which these expectations mediate nocebo effects.

In conclusion, this study demonstrates that nocebo effects can be effectively minimized by positive expectation induction and can even turn into placebo effects. Moreover, counterconditioning of nocebo effects regarding one stimulus can possibly generalize to another similar stimulus. Whereas more research is needed, the results of the current study show first indications that learning via counterconditioning and verbal suggestion may represent a promising strategy for diminishing nocebo responses.

## Supporting information

S1 Consort Checklist CONSORT 2010 checklist of information to include when reporting a randomised trial. (DOCX)

S1 Protocol Research protocol. (PDF)
