# Supplementary material for: Minimizing nocebo effects by conditioning with verbal suggestion: A randomized clinical trial in healthy humans
Source: PLoS One. 2017 Sep 14;12(9):e0182959. doi: 10.1371/journal.pone.0182959 (PMC5598922; doi:10.1371/journal.pone.0182959)
Supplement: S1 Protocol — (PDF) [file pone.0182959.s002.pdf]

RESEARCH PROTOCOL

(December 2014)

PROTOCOL TITLE

Can nocebo effects on itch be modified by a positive expectation induction?

**Protocol ID**	***<include protocol ID given by sponsor or investigator>***
**Short title**	**Can nocebo effects on itch be modified?**
**EudraCT number**	**n.a.**
**Version**	**2**
**Date**	**07-12 - 2014**
**Coordinating investigator/project leader**	**Prof. dr. A.W.M. Evers**<br>**Leiden University**<br>**Faculty of Social Sciences**<br>**Leiden, the Netherlands**<br>**Unit Health-, Medical & Neuropsychology**<br>**P.O. Box 9555**<br>**2300 RB Leiden**<br>**+31(0)71 527 6891**
**Principal investigator(s) (in Dutch: hoofdonderzoeker/ uitvoerder)**<br>***<Multicenter research: per site>***	**Prof. dr. A.W.M. Evers**
**Sponsor (in Dutch: verrichter/opdrachtgever)**	**Leiden University, Leiden, the Netherlands**
**Subsidising party**	**Netherlands Organization for Scientific Research (NWO)**
**Independent expert (s)**	**Dr. J.N. Bouwes Bavinck**<br>**Department of Dermatology**<br>**Leiden University Medical Center**<br>**PO Box 9600**<br>**2300 RC Leiden**<br>**+31(0)71 526 2421**

Laboratory sites <*if applicable*>	Prof. dr. B. Franke<br>Department of Human Genetics 855<br>Radboud university medical center<br>P.O. Box 910<br>6500 HB Nijmegen<br>+31 (0) 24 361 40 17
Pharmacy <*if applicable*>	C.M. van der Hulst<br>Trial manager Clinical Pharmacology & Toxicology<br>Leiden University Medical Center<br>Albinusdreef 2<br>2333 ZA Leiden<br>+31 (0)71 526 4285

PROTOCOL SIGNATURE SHEET

Name	Signature	Date
**Sponsor or legal representative:**<br>***<please include name and function>***<br><br>***<For non-commercial research,>***<br>**Head of Department:**<br>***<include name and function>***<br><br>**Prof. dr. A.W.M. Evers**<br>**Head of department**		
**[Coordinating Investigator/Project leader/Principal Investigator]:**<br>***<please include name and function>***<br><br>**Prof. dr. A.W.M. Evers**<br>**Principal Investigator**		

TABLE OF CONTENTS

1. INTRODUCTION AND RATIONALE 10
2. OBJECTIVES 12
3. STUDY DESIGN 13
4. STUDY POPULATION 15
4.1 Population (base) 15
4.2 Inclusion criteria 15
4.3 Exclusion criteria 15
4.4 Sample size calculation 15
5. TREATMENT OF SUBJECTS 16
5.1 Investigational product/treatment 16
5.2 Use of co-intervention (if applicable) 16
5.3 Escape medication (if applicable) 16
6. INVESTIGATIONAL PRODUCT 17
7. NON-INVESTIGATIONAL PRODUCT 18
8. METHODS 19
8.1 Study parameters/endpoints 19
8.1.1 Main study parameter/endpoint 19
8.1.2 Secondary study parameters/endpoints (if applicable) 19
8.1.3 Other study parameters (if applicable) 19
8.2 Randomisation, blinding and treatment allocation 19
8.3 Study procedures 19
8.4 Withdrawal of individual subjects 21
8.4.1 Specific criteria for withdrawal (if applicable) 21
8.5 Replacement of individual subjects after withdrawal 21
8.6 Follow-up of subjects withdrawn from treatment 21
8.7 Premature termination of the study 21
9. SAFETY REPORTING 22
9.1 Section 10 WMO event 22
9.2 AEs, SAEs and SUSARs 22
9.2.1 Adverse events (AEs) 22
9.2.2 Serious adverse events (SAEs) 22
9.2.3 Suspected unexpected serious adverse reactions (SUSARs) 23
9.3 Annual safety report 23
9.4 Follow-up of adverse events 23
9.5 [Data Safety Monitoring Board (DSMB) / Safety Committee] 23
10. STATISTICAL ANALYSIS 24
10.1 Primary study parameter(s) 24
10.2 Secondary study parameter(s) 24
10.3 Other study parameters 24
10.4 Interim analysis (if applicable) 24
11. ETHICAL CONSIDERATIONS 25

11.1 Regulation statement 25
11.2 Recruitment and consent 25
11.3 Objection by minors or incapacitated subjects (if applicable) 25
11.4 Benefits and risks assessment, group relatedness 25
11.5 Compensation for injury 25
11.6 Incentives (if applicable) 26
12. ADMINISTRATIVE ASPECTS, MONITORING AND PUBLICATION 27
12.1 Handling and storage of data and documents 27
12.2 Monitoring and Quality Assurance 27
12.3 Amendments 27
12.4 Annual progress report 27
12.5 End of study report 28
12.6 Public disclosure and publication policy 28
13. STRUCTURED RISK ANALYSIS 29
14. REFERENCES 30

LIST OF ABBREVIATIONS AND RELEVANT DEFINITIONS

ABR	ABR form, General Assessment and Registration form, is the application form that is required for submission to the accredited Ethics Committee (In Dutch, ABR = Algemene Beoordeling en Registratie)
AE	Adverse Event
AR	Adverse Reaction
CA	Competent Authority
CCMO	Central Committee on Research Involving Human Subjects; in Dutch: Centrale Commissie Mensgebonden Onderzoek
CV	Curriculum Vitae
DSMB	Data Safety Monitoring Board
EU	European Union
EudraCT	European drug regulatory affairs Clinical Trials
GCP	Good Clinical Practice
IB	Investigator’s Brochure
IC	Informed Consent
IMP	Investigational Medicinal Product
IMPD	Investigational Medicinal Product Dossier
METC	Medical research ethics committee (MREC); in Dutch: medisch ethische toetsing commissie (METC)
(S)AE	(Serious) Adverse Event
SPC	Summary of Product Characteristics (in Dutch: officiële productinfomatie IB1-tekst)
Sponsor	The sponsor is the party that commissions the organisation or performance of the research, for example a pharmaceutical company, academic hospital, scientific organisation or investigator. A party that provides funding for a study but does not commission it is not regarded as the sponsor, but referred to as a subsidising party.
SUSAR	Suspected Unexpected Serious Adverse Reaction
Wbp	Personal Data Protection Act (in Dutch: Wet Bescherming Persoonsgevens)
WMO	Medical Research Involving Human Subjects Act (in Dutch: Wet Medisch-wetenschappelijk Onderzoek met Mensen

SUMMARY

Rationale: For highly prevalent conditions associated with chronic itch, treatment effects are usually modest and vary strongly across patients. Expectancy mechanisms may contribute to this variability. The influence of expectations have often been studied in a placebo- or nocebo design, in which expectations are, for example, induced by verbal suggestions or a conditioning procedure. Placebo and nocebo effects can be defined as favorable and unfavorable treatment responses, unrelated to the treatment mechanism, which are induced by expectations of improvement and worsening, respectively. Placebo and nocebo effects have been investigated primarily in studies that focus on pain. Recent research of our research group showed in a validated design that expectancy mechanisms of verbal suggestion and conditioning can also induce placebo and nocebo effects on itch. However, it is not yet known whether nocebo effects can also actively be modified, e.g., by inducing a positive expectation induction, resulting in less itch. This is a scientifically and clinically highly relevant research question for the development of treatment modules to change inadequate negative expectations of patients suffering from chronic itch complaints.

Objective: The main objective of the study is to determine whether induced nocebo effects (negative expectancy effects) for itch can be modified by a positive expectation induction. Secondary objectives are to explore: a) the effects of expectation inductions on scratching behavior, b) the generalization of expectancy effects to other types of itch stimuli, c) the role of individual characteristics on expectancy effects, and d) the role of genetic predispositions on expectancy effects.

Study design: In healthy subjects, expectations with regard to electrically evoked itch will be induced by a conditioning with verbal suggestion procedure, in correspondence to a previous experiment conducted by the research group (25). For every stimulus, participants are asked to report the level of itch on a Visual Analogue Scale (VAS), and the scratching behavior of the participants will be recorded during the experiment. In part I, in all participants high-itch expectations will be induced (nocebo induction). More specifically, in the learning phase, short-lasting itch stimuli of medium and high intensity are repeatedly associated with certain colored cues displayed on a computer screen (e.g., two colored cues of which one cue is associated with itch stimuli of medium intensity (neutral cue), and the other with itch stimuli of high intensity (conditioned cue)). In line with the conditioning procedure, verbal suggestions for high itch will be given regarding itch stimuli associated with the conditioned cue. Subsequently, in the testing phase, expectancy effects for itch with regard to the colored cues will be tested by applying both the conditioned and neutral cues with itch stimuli of medium intensity. In part II, participants will be randomly assigned to one of three groups: the experimental group, in which low-itch expectations will be induced (group 1) (similar to the procedure described above with the exception that the conditioned cues are now associated with low itch intensity stimuli), a control group in which once more high-itch expectations will be induced similar to part I (group 2), or a control group with a neutral itch induction procedure (neutral expectation induction) (group 3). In part III, generalization of the induced expectancy effects will be tested with respect to another itch stimulus, i.e., histamine iontophoresis.

Study population: Healthy human volunteers, aged between 18 - 35 years old.

Intervention (if applicable): Not applicable.

Main study parameters/endpoints: The main study endpoint is the difference in the levels of itch (VAS scores) evoked by the electrical stimuli associated with the conditioned cues versus the neutral cues in the testing phase of part II of the experiment. It is investigated whether nocebo effects (negative expectation effects) can be modified by a positive expectation induction by conditioning and verbal suggestion (low-itch expectation induction) (group 1), resulting in lower itch VAS scores than a repeated negative expectation induction (high-itch expectation induction) (group 2), or a neutral procedure (neutral expectation induction) (group 3).

Nature and extent of the burden and risks associated with participation, benefit and group relatedness: Participants will complete a series of validated questionnaires at home to assess relevant individual characteristics, for approximately half an hour. Participants will then visit the department of Health-, Medical & Neuropsychology at the Leiden University once for approximately 5,5 hours. Sensations of itch will be induced using frequently applied, validated stimuli of short duration which are not burdensome (e.g., [1-4], 25). DNA is collected by asking participants to spit in a special tube. No risks are involved with participation in this study, only an investment of time.

1. INTRODUCTION AND RATIONALE

For highly prevalent conditions associated with chronic itch, treatment effects are usually modest and vary strongly between patients [5,6]. This treatment variability may partly be determined by patients' expectations regarding the treatment. A validated paradigm to experimentally study the influence of expectations is a placebo design, in which expectations are frequently induced by verbal suggestion or a conditioning procedure [7-10]. Placebo effects can be defined as favorable treatment responses, unrelated to the treatment mechanism, which are induced by expectations of improvement. Nocebo effects on the other hand, are unfavorable treatment responses, unrelated to the treatment mechanism, which are induced by expectations of worsening [9-11]. Placebo and nocebo effects have especially been investigated in studies that focus on pain. However, also with regard to itch, expectations seem to play an important role. There is some indirect evidence showing that patients with atopic dermatitis react differently to histamine when given verbal suggestions for exaggerated skin reactions and itch [12]. Furthermore, our research group showed experimentally that nocebo responses, and probably also placebo responses, can be induced on itch by verbal suggestions [3]. Moreover, results of our previous experiment for the first time indicated that the combination of conditioning with verbal suggestion is most effective to induce significant placebo and nocebo effects on itch [25]. However, at this point, it is still unknown whether nocebo effects (negative expectation effects) can actively be modified. This is an important research topic since nocebo effects can unfavorably affect clinical outcomes, can lead to a heightened report of side effects, and can negatively affect patients' daily health [9].

In addition to our main research objective, investigating the modifiability of the nocebo effect on itch, we also aim to provide insights in some secondary research topics. First, scratching behavior will additionally be measured, since itch is defined as an unpleasant sensation that can induce the urge to scratch [6,13]. Expectancy effects on itch VAS scores will be compared to the effects on scratching behavior. Furthermore, the possible extending of expectancy effects to other itch stimuli will be investigated, by assessing the generalization of expectancy effects regarding histamine iontophoresis. Additionally, individual characteristics may partly determine the magnitude of induced expectancy effects. For example, subjects scoring low on optimism are more likely to follow nocebo expectations [14]. Moreover, in our previous study on itch it was also found that individual characteristics (more negative affect, less extraversion, more neuroticism and less hope) were related to outcome expectancies, particularly with the nocebo effect [25]. Genotype is another factor that might affect individual differences in expectation responding. One relevant gene variant is the serotonin transporter length polymorphhic region (5-HTTLPR), for which two alleles have been identified, the short (s) and the long (l) allele. There is accumulating evidence that the 5-HTTLPR-s allele is associated with increased Pavlovian conditioning (e.g. [15,16]) and increased sensitivity to phrased descriptions of choice options in a decision making task [17]. Therefore, we hypothesize that 5-HTTLPR s-allele carriers may show increased responsivity to verbal suggestion and conditioning. Similarly, other

potentially relevant genetic variants will be tested for their involvement in these phenotypes, when possible relationships would emerge from literature.

The present research project aims to enhance the understanding of psychophysiological mechanisms of conditioning and verbal suggestion with regard to expectancy effects on itch. For the first time, the modifiability of negative expectancy effects will be examined systematically. Therefore, first negative expectations for itch will be induced as a starting point, which will subsequently be attempted to be reduced by a positive expectation induction. These effects will be compared with two control groups in which either once more negative expectations will be induced, or a neutral procedure will be applied. In addition, mechanisms of expectation responding are further explored with regard to the effects on scratching behavior; the generalization to other itch stimuli; and the influence of individual characteristics and genetic predispositions on individual expectation responding. The results of this study will enhance our understanding of the mechanisms underlying expectancy effects on itch and can provide insight into whether negative expectancy effects can be modified. These results may eventually help to develop therapeutic interventions by reducing inadequate negative expectations in patients suffering from chronic itch conditions. The results of this study can also be relevant to all kinds of physical symptoms and conditions other than itch, in which expectancy effects may play a role.

2. OBJECTIVES

Primary objective:

The primary objective of the study is to determine whether induced negative expectation effects for itch can be modified. It is hypothesized that levels of itch (VAS) will be reduced after a low-itch expectation induction, in comparison to the control procedures in which high-itch expectations or neutral expectations will be induced.

Secondary objectives:

Secondary goals are to explore:
a) the itch related expectancy effects on scratching behavior
b) the itch related expectancy effects regarding itch evoked by histamine iotophoresis
c) the role of individual characteristics (e.g., optimism) on (the modifiability of) expectancy effects
d) the role of 5-HTTLPR genotype and other genetic variants on (the modifiability of) expectancy effects

3. STUDY DESIGN

Participants will complete online validated questionnaires at home which will take about 30 minutes, and will visit the department of Health, Medical & Neuropsychology of the Leiden University once, for approximately 5,5 hours. The experiment will take place in a research lab of the department.

The experiment consists of three parts (see Fig. 1 for an overview of the components of the study). In part I and II, expectations with regard to electrically evoked itch will be induced by conditioning with verbal suggestion. Individual itch thresholds are used for determining the intensity of the itch stimuli for the conditioning procedure. In part I of the experiment, all participants will be subjected to a high-itch expectation induction, in order to attain negative expectations with regard to itch as a starting point. In part II of the experiment, participants are assigned to either the experimental group, in which low-itch expectations will be induced (group 1), or one of the control groups in which either once more high-itch expectations will be induced (group 2), or neutral expectations (group 3). Participants will be asked to report their level of itch for every stimulus on a Visual Analogue Scale (VAS), and scratching behavior of the participants will be recorded during the experiment. In part III, histamine iontophoresis will be applied (with the conditioned colored cue displayed on the computer screen) and in addition participants are asked to spit in a special tube to collect saliva for DNA isolation.

Expectation induction procedures

Part I

High-itch expectation induction (all participants)

High-itch expectations will be induced in all participants regarding electrical stimuli by conditioning with verbal suggestion. A conditioning procedure will be applied in which a specific colored cue displayed on a computer screen is repeatedly associated with an increased intensity itch stimulus (conditioned cue). In line with the conditioning procedure, verbal suggestions for high itch will be given regarding itch stimuli associated with the conditioned cue.

Part II

Group 1: Low-itch expectation induction

Participants in this group will receive a low-itch expectation induction regarding electrical stimuli by conditioning with verbal suggestion. A conditioning procedure will be applied in which the same specific colored cue as described above displayed on a computer screen (conditioned cue) is repeatedly associated with a decreased intensity of the itch stimulus. In line with the conditioning procedure, verbal suggestions for low itch will be given regarding the itch stimuli.

Group 2: High-itch expectation induction

Participants in this group will receive the same high-itch expectation induction as in part I, described above in part I.

Group 3: Neutral expectation induction

Participants in this group will not receive a conditioning procedure or verbal suggestions. The two different colored cues will be shown with randomly applied itch stimuli and participants are merely told that the colored cues will indicate the start of a new stimulus.

Fig 1. Overview of the study

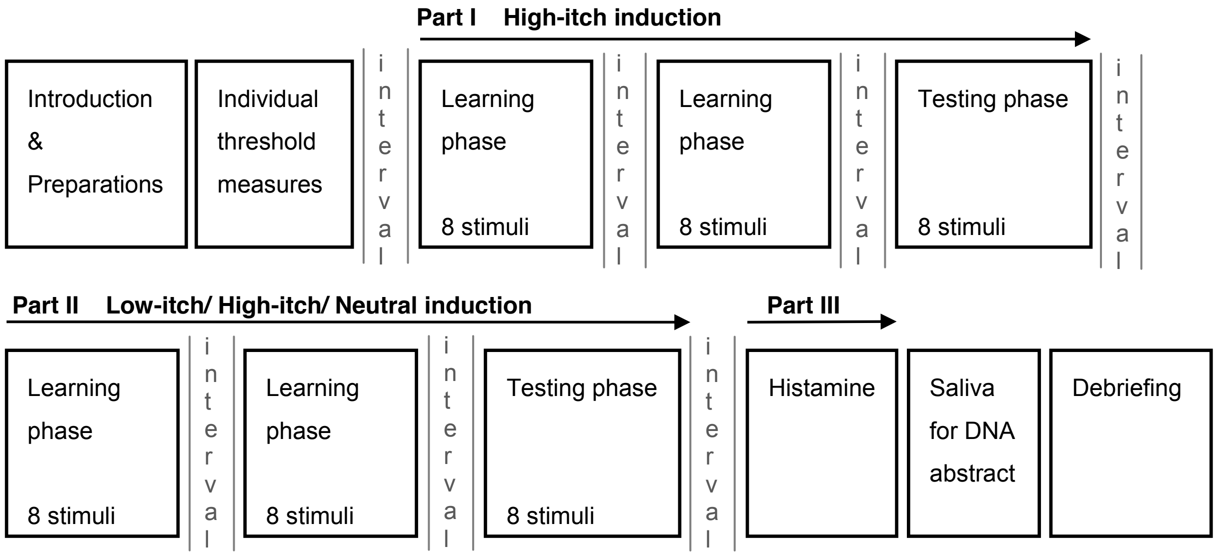

4. STUDY POPULATION

4.1 Population (base)

Healthy participants aged 18 – 35 years will be included in the study. Participants will be recruited via different approaches.

4.2 Inclusion criteria

Healthy human volunteers, 18 - 35 year old, fluent in Dutch language.

4.3 Exclusion criteria

Severe morbidity (e.g., multiple sclerosis, diabetes mellitus, heart or lung diseases), psychiatric disorders (e.g., depression), use of pacemaker, color-blindness, diagnose of histamine hypersensitivity, and chronic itch or pain complaints.

4.4 Sample size calculation

The required sample size was calculated, in agreement with a statistician (R. Donders, Radboudumc), based on our previous study with a comparable design [25]. Such as in the present study, in our previous study a comparison was made between a positive expectation induction by conditioning and verbal suggestion and a control group. In this study an average placebo effect of 0.86 (SD = 0.59) and 0.32 (SD = 0.79) was found in the *conditioning with verbal suggestion group* and the *control group,* respectively (effect size of d = 0.78). Based on these means and standard deviations, sample size calculations were done using G*power 3.1. We simplified the analyses of the main hypotheses, Univariate analyses of variance (ANOVA's) with Dunnett contrasts, into two two-tailed independent samples t-tests (i.e., comparing the positive expectation induction group with the negative expectation induction group and the neutral expectation induction group separately) using a Bonferroni correction. For these t-tests, the means and standard deviations of the most conservative comparison in the present study were used, i.e., of the positive expectation induction with the neutral expectation induction. With an effect size of d = 0.78, an alpha of 0.025 and a desired power of 0.80, this resulted in an estimated total sample size of 33 participants per group.

5. TREATMENT OF SUBJECTS

< This chapter is only applicable for intervention studies>

5.1 Investigational product/treatment

Not applicable

5.2 Use of co-intervention (if applicable)

Not applicable.

5.3 Escape medication (if applicable)

Not applicable.

6. INVESTIGATIONAL PRODUCT

<This chapter is applicable for research with any product; medicinal product, food product, medical device or other>

Not applicable.

7. NON-INVESTIGATIONAL PRODUCT

<This chapter is applicable for any other product that is used in the study, like challenge agents or products used to asses end-points in the trial. This can be a medicinal product or a food product or a chemical compound or stable isotope or other product.

*This chapter does **not** include co-medication or escape medication, these are already mentioned in chapter 5*

For products to be used as in usual clinical practice the information can be limited to the chapters 7.1, 7.6 and 7.7 >

Not applicable.

8. METHODS

8.1 Study parameters/endpoints

8.1.1 Main study parameter/endpoint

The main study endpoint is the level of itch (VAS scores) evoked by the electrical stimuli associated with the conditioned cues in comparison to the neutral cues in the testing phase of part II of the experiment. It is investigated whether nocebo effects (negative expectation effects) can be modified by a positive expectation induction by conditioning and verbal suggestion (low-itch expectation induction) (group 1), resulting in lower itch VAS scores than a repeated negative expectation induction (high-itch expectation induction) (group 2), or neutral procedure (neutral expectation induction) (group 3).

8.1.2 Secondary study parameters/endpoints

The secondary study endpoints of the present experiment are: a) the scratching behavior of the participants, b) the levels of itch (VAS ratings) induced by histamine iontophoresis after displaying the conditioned cue, c) the scores on several questionnaires measuring individual characteristics related to expectancy effects, d) the 5-HTTLPR genotype and possibly also other genetic variants.

8.1.3 Other study parameters (if applicable)

Not applicable.

8.2 Randomisation, blinding and treatment allocation

After part I, in which all participants are exposed to the same procedure, in part II participants will be randomly allocated to one of three experimental groups (see section *3. Study design* and *Fig 1.*). We will use a single blind design: Since the instructions given to the subjects differ in accordance with the group the subjects are allocated to, only subjects will be blinded for the randomization to different groups. No indications for breaking the randomization code are indicated.

8.3 Study procedures

Expectation induction

Expectations will be induced by conditioning and verbal suggestion procedures with regard to two specific colored cues being displayed on a computer screen. For half of the participants the first colored cue is the conditioned cue and the second colored cue is the neutral cue, and visa versa for the remaining participants. In part I of the experiment the high- itch expectation induction procedure, as described below, is applied for all participants. In part II, participants are allocated to one of the three experimental groups in which either the low-, high-, or neutral expectation induction is applied (see section *3. Study design* and *Fig 1.*).

<u>High-itch expectation induction</u>

For the high-itch expectation induction, the intensity (mA) of the stimuli will be increased following the presentation of the conditioned cue (*expectation for high itch*) in comparison to the intensity of stimuli following the neutral cue, which is not adjusted (*neutral expectation*). In line, participants will be told that: “The specific colored cue will signal the activation of the third electrode that induces an increase in the intensity of the itch stimulus *(expectation for high itch)*. The other cue will indicate that the third electrode is turned off and will not change the intensity of the itch stimulus *(neutral expectation)”*.

Low-itch expectation induction

For the low-itch expectation induction, the intensity (mA) of the stimuli will be decreased following the presentation of the conditioned cue (*expectation for low itch*) in comparison to the intensity of stimuli following the neutral cue, which is not adjusted (*neutral expectation*). In line, participants will be told that: “The specific colored cue will signal the activation of the third electrode that induces a decrease in the intensity of the itch stimulus *(expectation for low itch)*. The other cue will indicate that the third electrode is turned off and will not change the intensity of the itch stimulus *(neutral expectation)”*.

Neutral expectation induction

In the neutral group, participants will not receive a conditioning procedure or verbal suggestions; the different colored lights are randomly shown with previously defined random stimuli intensities and participants are merely told that a colored cue will indicate the start of a new stimulus.

Participants are asked to rate their levels of itch on a Visual Analogue Scale (VAS) ranging from 0 (no itch at all) to 10 (worst itch imaginable) after each stimulus. Additionally, participants are videotaped to record scratching behavior of the participants during the experimental sessions.

Somatosensory stimuli

Itch sensations are induced by two cutaneous electrodes applied on the forearm. A third and inactive electrode is placed between the two active electrodes and represents the sham treatment designed to induce expectations of stimulus alterations. Itch thresholds will be determined according to the protocol of our previous study [25], which was based on the protocol of Colloca [18,19], to apply stimuli with strengths adjusted to participant’s individual sensitivity levels. This method showed good validity in our previous study on itch [25]. These threshold measures will be used to adjust the intensity (mA) of the stimuli for conditioning of expectations with regard to itch.

In addition, histamine iontophoresis, previously applied by our research group [1-3,20,21], will be applied to test possible generalization of expectancy effects to other types of itch stimuli. A 0.3% diphosphate histamine solution will be used in the present study. Each participant will undergo one histamine application at the inner side of the forearm, while the conditioned cue (group 1 and 2) or a neutral cue (group 3) will be presented on the computer screen.

Questionnaires for individual characteristics

Several validated questionnaires that have previously been related to expectancy learning mechanisms will be completed by the participants to assess the role of individual characteristics on expectancy effects on itch [3,14,24].

Genotype

DNA will be isolated from the saliva samples collected in Oragene tubes and 5-HTTLPR (variable number tandem repeat (VNTR)) genotyping will be conducted by PCR and subsequent analysis of product lengths, as we performed previously (CMO registration number 2010/ 450). Additional genotypes will be tested on the longer term, by either classical genotyping or next generation sequencing techniques.

Debriefing

Participants will be debriefed after completion of the experiment and informed about the purpose of the study. Participants will be given the opportunity to ask questions they have regarding the experiment and their participation in it.

8.4 Withdrawal of individual subjects

Subjects can leave the study at any time for any reason if they wish to do so without any consequences. The investigator can decide to withdraw a subject from the study for urgent medical reasons.

8.4.1 Specific criteria for withdrawal (if applicable)

Not applicable.

8.5 Replacement of individual subjects after withdrawal

As in our previous studies, in which withdrawal of subjects was highly uncommon [e.g., 3, 25], also in the present study low withdrawal is expected (e.g., due to apparatus not functioning properly). Participants who have withdrawn from the study or for which data loss will occur, will be replaced by randomly selected additional participants with a maximum 10%.

8.6 Follow-up of subjects withdrawn from treatment

Not applicable.

8.7 Premature termination of the study

Not applicable.

9. SAFETY REPORTING

9.1 Section 10 WMO event

In accordance to section 10, subsection 1, of the WMO, the investigator will inform the subjects and the reviewing accredited METC if anything occurs, on the basis of which it appears that the disadvantages of participation may be significantly greater than was foreseen in the research proposal. The study will be suspended pending further review by the accredited METC, except insofar as suspension would jeopardise the subjects' health. The investigator will take care that all subjects are kept informed.

9.2 AEs, SAEs and SUSARs

9.2.1 Adverse events (AEs)

Adverse events are defined as any undesirable experience occurring to a subject during the study, whether or not considered related to [the investigational product / the experimental interventiont]. All adverse events reported spontaneously by the subject or observed by the investigator or his staff will be recorded.

9.2.2 Serious adverse events (SAEs)

A serious adverse event is any untoward medical occurrence or effect that at any dose:

- results in death;
- is life threatening (at the time of the event);
- requires hospitalisation or prolongation of existing inpatients' hospitalisation;
- results in persistent or significant disability or incapacity;
- is a congenital anomaly or birth defect;
- Any other important medical event that may not result in death, be life threatening, or require hospitalization, may be considered a serious adverse experience when, based upon appropriate medical judgement, the event may jeopardize the subject or may require an intervention to prevent one of the outcomes listed above.

The sponsor will report the SAEs through the web portal *ToetsingOnline* to the accredited METC that approved the protocol, within 15 days after the sponsor has first knowledge of the serious adverse reactions.

SAEs that result in death or are life threatening should be reported expedited. The expedited reporting will occur not later than 7 days after the responsible investigator has first knowledge of the adverse reaction. This is for a preliminary report with another 8 days for completion of the report.

9.2.3 Suspected unexpected serious adverse reactions (SUSARs)

Not applicable.

9.3 Annual safety report

Not applicable.

9.4 Follow-up of adverse events

All AEs will be followed until they have abated, or until a stable situation has been reached. Depending on the event, follow up may require additional tests or medical procedures as indicated, and/or referral to the general physician or a medical specialist.

SAEs need to be reported till end of study within the Netherlands, as defined in the protocol

9.5 [Data Safety Monitoring Board (DSMB) / Safety Committee]

Not applicable.

10. STATISTICAL ANALYSIS

10.1 Primary study parameter(s)

All variables are continuous. Descriptive statistics (means, standard deviations, etc.) will be calculated of all relevant variables. Variables will be checked for outliers and skewness as these can severely limit the usefulness of the mean as measure for location.

In consultation with the statistician associated with the study (R. Donders), our main hypothesis, that the induced levels of itch (VAS) will be lower after a positive expectation induction (low-itch expectation induction) (group 1), in comparison to a negative expectation induction (high-itch expectation induction) (group 2), or neutral expectation induction (group 3), will be tested in an ANOVA using Dunnett contrasts for comparing the expectancy effect on itch in group 1 with the effect in group 2 and group 3.

10.2 Secondary study parameter(s)

Similarly to the primary outcome measure, ANOVAs will be conducted for the secondary outcome measures with regard to expectancy effects on histamine evoked itch, scratching behavior, and genotype. Additionally, Pearson correlation coefficients will be calculated between the individual characteristics and the expectancy effects on itch.

10.3 Other study parameters

Not applicable.

10.4 Interim analysis (if applicable)

Not applicable.

11. ETHICAL CONSIDERATIONS

11.1 Regulation statement

The study will be conducted according to the principles of the Declaration of Helsinki (21-10-2008) and in accordance with the Medical Research Involving Human Subjects Act (WMO).

11.2 Recruitment and consent

Healthy participants will be recruited by advertisement. When subjects show their interest in participating, they will receive adequate written information regarding the study. Subjects will be given one week to consider their decision to participate in the study. When participants arrive at the Leiden University for the day of testing, they will be informed orally about the study and sign the informed consent.

11.3 Objection by minors or incapacitated subjects (if applicable)

Not applicable.

11.4 Benefits and risks assessment, group relatedness

Although no direct benefits are expected to be experienced by the participants, no risks are involved with participation in this study. The only burden for participants in the investment of time.

11.5 Compensation for injury

The sponsor/investigator has a liability insurance which is in accordance with article 7, subsection 6 of the WMO.

The sponsor (also) has an insurance which is in accordance with the legal requirements in the Netherlands (Article 7 WMO and the Measure regarding Compulsory Insurance for Clinical Research in Humans of 23th June 2003). This insurance provides cover for damage to research subjects through injury or death caused by the study.

1. € 450.000,-- (i.e. four hundred and fifty thousand Euro) for death or injury for each subject who participates in the Research;

2. € 3.500.000,-- (i.e. three million five hundred thousand Euro) for death or injury for all subjects who participate in the Research;

3. € 5.000.000,-- (i.e. five million Euro) for the total damage incurred by the organisation for all damage disclosed by scientific research for the Sponsor as 'verrichter' in the meaning of said Act in each year of insurance coverage.

The insurance applies to the damage that becomes apparent during the study or within 4 years after the end of the study.

A request for dispensation of liability and participants insurance is requested, since participation in the study is without risks other than investment of time (see procedure and methods). This is in line with the medical ethical review committee evaluation of our previous studies (e.g., CMO registration number 2010/ 450).

11.6 Incentives (if applicable)

Participants will receive a small monetary reimbursement (€35) and will be compensated for travelling costs (when the travelling distance is > 15 km).

12. ADMINISTRATIVE ASPECTS, MONITORING AND PUBLICATION

12.1 Handling and storage of data and documents

Anonymous participant identification codes will be used to link data to participants. The file containing the linking between participant numbers and personal data (e.g., name, date of birth) will be managed by the researchers and data manager and will be locked for access by others. Collected data (e.g., questionnaires, laboratory results, informed consents) will be stored for a period of 15 years.

12.2 Monitoring and Quality Assurance

According to the standard procedures of the data manager, the anonymity of the personal data will be guaranteed and data entry will be checked regularly (the current data monitoring protocol of the department of medical psychology of the Radboudumc will be used). The monitor of the study will later be appointed.

12.3 Amendments

Amendments are changes made to the research after a favourable opinion by the accredited METC has been given. All amendments will be notified to the METC that gave a favourable opinion.

All amendments will be notified to the METC that gave a favourable opinion. Non-substantial amendments will not be notified to the accredited METC and the competent authority, but will be recorded an filed by the sponsor.

A ‘substantial amendment’ is defined as an amendment to the terms of the METC application, or to the protocol or any other supporting documentation, that is likely to affect to a significant degree:

- the safety or physical or mental integrity of the subjects of the trial;
- the scientific value of the trial;
- the conduct or management of the trial; or
- the quality or safety of any intervention used in the trial.

All substantial amendments will be notified to the METC and to the competent authority.

Non-substantial amendments will not be notified to the accredited METC and the competent authority, but will be recorded and filed by the sponsor.

12.4 Annual progress report

The sponsor/investigator will submit a summary of the progress of the trial to the accredited METC once a year. Information will be provided on the date of inclusion of the first subject, numbers of subjects included and numbers of subjects that have completed the trial, serious adverse events/ serious adverse reactions, other problems, and amendments.

12.5 End of study report

The investigator will notify the accredited METC of the end of the study within a period of 8 weeks. The end of the study is defined as the last patient's last visit. In case the study is ended prematurely, the investigator will notify the accredited METC within 15 days, including the reasons for the premature termination. Within one year after the end of the study, the investigator/sponsor will submit a final study report with the results of the study, including any publications/abstracts of the study, to the accredited METC.

The sponsor will notify the accredited METC and the competent authority of the end of the study within a period of 90 days. The end of the study is defined as the last patient's last visit.

In case the study is ended prematurely, the sponsor will notify the accredited METC and the competent authority within 15 days, including the reasons for the premature termination.

Within one year after the end of the study, the investigator/sponsor will submit a final study report with the results of the study, including any publications/abstracts of the study, to the accredited METC and the Competent Authority.

<*In case the final study report will not be available within one year, another term should be defined including the reasons.*>

12.6 Public disclosure and publication policy

<Please mentionon the arrangements made between the sponsor and the investigator concerning the public disclosure and publication of the research data.>

In accordance with the CCMO statement on publication policy, the results of this study will be disclosed unreservedly, i.e., regardless of confirmation or disconfirmation of the hypotheses. The results will be submitted for publication in peer-reviewed journals.

STRUCTURED RISK ANALYSIS

Not applicable.

12.7 Synthesis

Not applicable.

13. REFERENCES

1. van Laarhoven AI, Kraaimaat FW, Wilder-Smith OH, van Riel PL, van de Kerkhof PC, et al. (2013) Sensitivity to itch and pain in patients with psoriasis and rheumatoid arthritis. Exp Dermatol 22: 530-534.
2. van Laarhoven AI, Walker AL, Wilder-Smith OH, Kroeze S, van Riel PL, et al. (2012) Role of induced negative and positive emotions in sensitivity to itch and pain in women. Br J Dermatol 167: 262-269.
3. van Laarhoven AI, Vogelaar ML, Wilder-Smith OH, van Riel PL, van de Kerkhof PC, et al. (2011) Induction of nocebo and placebo effects on itch and pain by verbal suggestions. Pain 152: 1486-1494.
4. van Laarhoven AI, Kraaimaat FW, Wilder-Smith OH, Evers AW (2010) Role of attentional focus on bodily sensations in sensitivity to itch and pain. Acta Derm Venereol 90: 46-51.
5. Biro T, Ko MC, Bromm B, Wei ET, Bigliardi P, et al. (2005) How best to fight that nasty itch - from new insights into the neuroimmunological, neuroendocrine, and neurophysiological bases of pruritus to novel therapeutic approaches. Exp Dermatol 14: 225-240.
6. Yosipovitch G, Greaves MW, Schmelz M (2003) Itch. Lancet 361: 690-694.
7. Benedetti F, Amanzio M (2011) The placebo response: How words and rituals change the patient's brain. Patient Education and Counseling 84: 413-419.
8. Colloca L, Klinger R, Flor H, Bingel U (2013) Placebo analgesia: Psychological and neurobiological mechanisms. Pain 154: 511-514.
9. Colloca L, Miller FG (2011) The nocebo effect and its relevance for clinical practice. Psychosomatic Medicine 73: 598-603.
10. Price DD, Finniss DG, Benedetti F (2008) A comprehensive review of the placebo effect: Recent advances and current thought. Annual Review of Psychology 59: 565-590.
11. Benedetti F (2008) Mechanisms of placebo and placebo-related effects across diseases. Annual Review of Pharmacology and Toxicology 48: 33-60.
12. Scholz O, Hermanns N (1994) Illness behavior and cognitions influence the perception of itching of patients suffering from atopic dermatitis. Z Klin Psychol 23: 127-135.
13. Papoiu AD, Wang H, Coghill RC, Chan YH, Yosipovitch G (2011) Contagious itch in humans: a study of visual 'transmission' of itch in atopic dermatitis and healthy subjects. Br J Dermatol 164: 1299-1303.
14. Geers AL, Helfer SG, Kosbab K, Weiland PE, Landry SJ (2005) Reconsidering the role of personality in placebo effects: dispositional optimism, situational expectations, and the placebo response. J Psychosom Res 58: 121-127.
15. Klucken T, Alexander N, Schweckendiek J, Merz CJ, Kagerer S, et al. (2013) Individual differences in neural correlates of fear conditioning as a function of 5-HTTLPR and stressful life events. Soc Cogn Affect Neurosci 8: 318-325.
16. Lonsdorf TB, Weike AI, Nikamo P, Schalling M, Hamm AO, et al. (2009) Genetic gating of human fear learning and extinction: possible implications for gene-environment interaction in anxiety disorder. Psychol Sci 20: 198-206.
17. Roiser JP, de Martino B, Tan GC, Kumaran D, Seymour B, et al. (2009) A genetically mediated bias in decision making driven by failure of amygdala control. Journal of Neuroscience 29: 5985-5991.
18. Colloca L, Petrovic P, Wager TD, Ingvar M, Benedetti F (2010) How the number of learning trials affects placebo and nocebo responses. Pain 151: 430-439.
19. Colloca L, Sigaudo M, Benedetti F (2008) The role of learning in nocebo and placebo effects. Pain 136: 211-218.
20. van Laarhoven AI, Kraaimaat FW, Wilder-Smith OH, van de Kerkhof PC, Evers AW (2010) Heterotopic pruritic conditioning and itch--analogous to DNIC in pain? Pain 149: 332-337.
21. van Laarhoven AI, Kraaimaat FW, Wilder-Smith OH, van de Kerkhof PC, Cats H, et al. (2007) Generalized and symptom-specific sensitization of chronic itch and pain. J Eur Acad Dermatol Venereol 21: 1187-1192.
24. Geers AL, Kosbab K, Helfer SG, Weiland PE, Wellman JA (2007) Further evidence for individual differences in placebo responding: an interactionist perspective. J Psychosom Res 62: 563-570.

25. Bartels et al. Role of conditioning and verbal suggestion in placebo and nocebo effects on itch. Manuscript submitted for publication.
